# Dermatomyositis Sine Myositis with Membranoproliferative Glomerulonephritis

**DOI:** 10.1155/2012/751683

**Published:** 2012-08-13

**Authors:** Mohammad Bagher Owlia, Roya Hemayati, Shokouh Taghipour Zahir, Mohammad Moeini Nodeh

**Affiliations:** ^1^Department of Rheumatology, Shahid Sadoughi University of Medical Sciences, Yazd 89168-86938, Iran; ^2^Department of Nephrology, Shahid Sadoughi University of Medical Sciences, Yazd 89168-86938, Iran; ^3^Department of Pathology, Shahid Sadoughi University of Medical Sciences, Yazd 89168-86938, Iran; ^4^Department of Internal Medicine, Shahid Sadoughi University of Medical Sciences, Yazd 89168-86938, Iran

## Abstract

Dermatomyositis (DM) is an autoimmune disease that is characterized by involvement of proximal musculature and skin. We report a 52-year-old woman with a 6-year history of dermatomyositis sine myositis, who developed lower extremity edema and proteinuria. Pathological examination of renal biopsy showed membranoproliferative glomerulonephritis. She received steroid, cyclophosphamide, and mycophenolate mofetil. Over the 9 to 10 months after the beginning of treatment, the proteinuria was improved.

## 1. Introduction

Dermatomyositis (DM) is an autoimmune disease that characterized by involvement of proximal musculature and skin. Rarely DM presents with characteristic cutaneous manifestations without muscle involvement, the so called dermatomyositis sine myositis [1]. Renal involvement is uncommon in dermatomyositis especially in sine myositis variant of DM compared to other autoimmune disorders such as SLE, systemic scleroderma, and microscopic polyarteritis nodosa [2]. We report a patient who developed membranoproliferative glomerulonephritis (MPGN) 6 years after the diagnosis of dermatomyositrs sine myositis.

## 2. Case Report

A 52-year-old woman was admitted to our hospital with chronic cough, exertional dyspnea, and edema of lower extremities in November 2010. Dermatomyositis sine myositis was diagnosed for her with typical Gottron's sign, heliotrope rash, episodes of mechanic's hand, and nail fold capillary changes without muscle weakness about 6 years ago. At that time, serologic tests such as ANA and anti-dsDNA were negative, and C3, C4, and CH50 levels were normal. Her ischemic ulcers on hand knuckles showed infiltrations of lymphoplasma cells without evidence of overt vasculitis on biopsy specimens in favor of nonspecific connective tissue diseases. 

In April 2004, She developed fever, cough, diarrhea, and vomiting, while she had been treated with prednisolone and azathioprine. After a course of antibiotic therapy and due to cytopenia, azathioprine switched to cyclosporine. Six years later, the patient had several hospital admissions because of dry cough, exertional dyspnea, and fever. Initially, computed tomography (CT) of the chest and echocardiography were normal. However, later pulmonary function tests revealed restrictive pattern and high-resolution CT scan of the lungs showed fibrosis of anterior segment of the right upper lobe. She developed diabetes mellitus and hypertension 3 years ago.

The patient was taking prednisolone, cyclosporine, hy droxychloroquine, amlodipine, triamterene, hydrochlorothiazide, aspirin, and glybenclamide, without kidney involvement over the past 6 years. She never used any immunosuppressive drugs before the diagnosis of dermatomyositis sine myositis.

On last admission, body temperature of 37°C and blood pressure of 160/85 mmHg were recorded. Physical exam revealed heliotrope rash in upper eyelids, coarse crackles over the base of the right lung, and pitting edema of lower extremities. Laboratory findings were as follows: mild anemia Hemoglobin 11.1 g/dL (12–16 g/dL), White blood cell 7900 cells/mm^3^ (3500–10500  cells/mm^3^), blood urea nitrogen 28 mg/dL (7–20 mg/dL), creatinine 1 mg/dL (0.5–0.9 mg/dL), 24-hours creatinine clearance 83 cc/min, triglyceride 325 mg/dL (30–200 mg/dL), total cholesterol 340 mg/dL (<200 mg/dL), high-density lipoprotein 68 mg/dL (40–60 mg/dL), low-density lipoprotein 207 mg/dL (<130 mg/dL), and abnormal urinalysis with 2+ proteinuria; urinary protein excretion level was 2.2 g/day. The serum creatine kinase (CK), lactate dehydrogenase (LDH), alanine aminotransferase (ALT), and aspartate aminotransferase (AST) levels were 34 IU/L (39–238 IU/L), 579 IU/L (100–500 IU/L), 23 IU/L (7–41 IU/L), and 16 IU/L (12–38 IU/L), respectively. Erythrocyte sedimentation rate (ESR) was substantially elevated 75 mm/hour and C-reactive protein was 2+ positive. Anti-dsDNA, p-ANCA, and c-ANCA were negative. Complement levels (C3, C4, and CH50) were within normal range. Serologic tests for hepatitis B and C were negative. Later assays showed positive fluorescent ANA result with homogeneous pattern. But anti-Jo1 test was not available in our center at the time of primary investigations.

**Figure 1:**
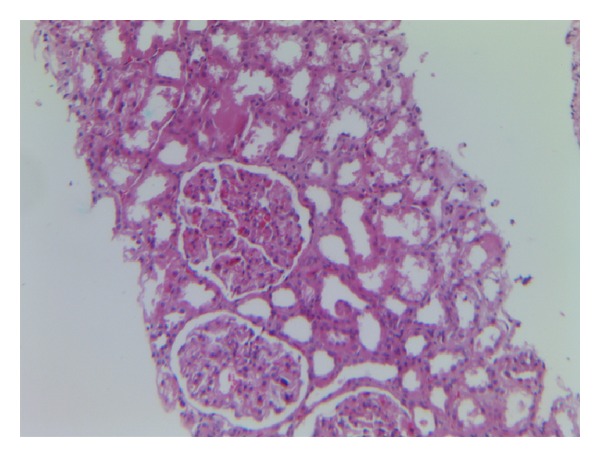
Glomeruli are large and hypercellular with proliferation of cells in the mesangium and leukocytic infiltration.

**Figure 2:**
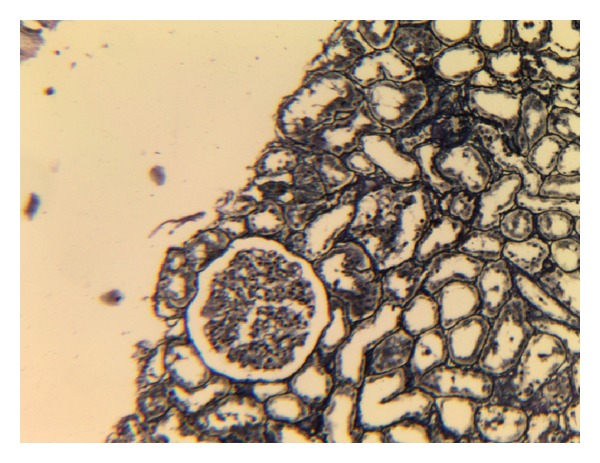
MPGN with mesangial cell proliferation, an increase in mesangial matrix (black staining in silver stain) and basement membrane thickening and tram track appearance in silver staining.

A percutaneous renal biopsy was performed. Microscopic examination revealed diffuse enlargement of glomeruli with thickening of the capillary walls, thickened mesangial matrix, mesangial cells proliferation causing lobulation of the tufts, and scattered infiltration of neutrophils (Figure 1). The capillary wall had tram-track appearance in silver staining (Figure 2). The interstitium had no significant pathological changes. Immunofluorescence showed granular pattern of C3 and IgG deposition along the capillary walls and mesangial matrix. Based on biopsy results, diagnosis of membranoproliferative glomerulonephritis was made.

On last admission, the patient was treated with 500 mg intravenous methylprednisolone and 1000 mg cyclophosphamide for 3 subsequent months. She started to get 2000 mg mycophenolate mofetil and 30 mg prednisolone daily. After 10 months of beginning treatment, 24-hour protein excretion was decreased to less than 150 mg/day. 

## 3. Discussion

Most of autoimmune disorders have common or similar mechanisms involving mostly collagen containing tissues all over the body. Associations of specific organ involvement in each specific connective tissue diseases are well known. In classic forms, dermatomyositis is diagnosed with classic features of skin disease (Gottron's papules, mechanic's hand, and heliotrope rash) along with clinical and laboratory evidence of inflammatory muscle disease. Involvement of skin and lungs along with striated and cardiac muscles are well established in classic dermatomyositis. Dermatomyositis sine myositis is a rather rare subtype of dermatomyositis with prominent typical skin lesions of dermatomyositis without clinical or laboratory finding of myositis. Pulmonary involvement frequently is seen in this group. While kidney involvement is one of the major problems in cases of some connective tissue disease such as systemic lupus erythematosus (SLE), renal disease in dermatomyositis is uncommon. The following types of involvement are mainly described in dermatomyositis: renal tubular damage associated with myoglobinemia and myoglobinuria following acute rhabdomyolysis [2]. Chronic glomerulonephritis is quite rare in dermatomyositis. To date, based on our search, only 8 cases of documented glomerulonephritis have been reported [3–10]. In four cases dermatomyositis and glomerulonephritis developed concurrently and in the rest of cases glomerulonephritis presented 1.5–9 years after the diagnosis of dermatomyositis. In all cases, levels of proteinuria were above 1 g/day. Renal biopsies were suggestive of membranous nephritis (5 cases), MPGN (2 cases), and diffuse proliferative glomerulonephritis (1 case). Immunofluorescence staining reveals positivity for immunoglobuline, and complements in all cases.

In our patient, MPGN emerged after 6 years of developing dermatomyositis sine myositis, with 2.2 g protein excretion in 24 hour. Ophthalmologic examination ruled out diabetic and hypertensive retinopathy. Serologic tests for hepatitis B and C, lupus, and systemic vasculitides were negative. Serum complement levels were normal. While approximately 75% of MPGN patients have depressed complement level due to activation of alternative complement pathway, [11] but it is not a consistent feature [12]. This case could be categorized as “normal complement MPGN”. Negative ANA results are against the diagnosis of dermatomyositis but considering lower sensitivity of commercial available enzyme linked immunosorbent (ELISA) assays for ANA, the first negative results are offset with later positive fluorescent ANA test. Positive extractable nuclear antigens (ENA) albeit supporting and of use to discriminate and prognosticate subtypes of dermatomyositis, but not essential for diagnosis. Typical skin lesions of dermatomyositis along with nail fold capillary changes in a pertinent clinical setting are almost always pathognomonic for dermatomyositis.

In conclusion, our case is the first description of dermatomyositis sine myositis with MPGN.
